# New trend in colorectal cancer in Germany: are young patients at increased risk for advanced colorectal cancer?

**DOI:** 10.1186/s12957-017-1227-z

**Published:** 2017-08-23

**Authors:** Peter C. Ambe, Stefan Jansen, Hubert Zirngibl

**Affiliations:** Department of Surgery, Helios University Hospital Wuppertal, Witten-Herdecke University, Heusnerstr. 40, 42283 Wuppertal, Germany

**Keywords:** Colorectal cancer, Young age, Screening colonoscopy, Overall survival, CME, TME

## Abstract

**Background:**

The role of colonoscopy in the screening of colorectal cancer (CRC) has been unequivocally established. In Germany, screening colonoscopy with full insurance reimbursement is available for individuals aged 55 and above, and/or for persons with well-known risk factors for CRC. However, advanced CRC is not uncommon in individuals below 55 years. This study was designed to investigate the incidence of advanced CRC in patients < 55 years.

**Methods:**

A retrospective analysis of data from a prospectively maintained CRC database of a university hospital in Germany was performed. Using the recommended age for screening colonoscopy as cutoff, the study population was divided into two groups: < 55 years (study group) and ≥ 55 years (control group). Both groups were compared with regard to the extent of CRC using the UICC stages. Only surgically managed patients were included for analysis. Advanced CRC was defined as UICC stage III or IV.

**Results:**

Complete follow-up data was available for 609 patients treated between 2009 and 2013. The study group included 83 patients, 42 females and 41 males with a median age of 48.0 ± 10 years, while the control group was made up of 526 patients, 230 females and 296 males with a median age of 75.5 ± 8.3 years. Both groups were comparable with regard to gender distribution, *p* = 0.24. Significantly more patients from the study group were diagnosed with advanced CRC in comparison to the control group, 56.6 vs. 43.9%, *p* = 0.03. There was no statistically significant difference amongst both groups with respect to cancer-related mortality, 10.8 vs. 12.5%, *p* = 0.66.

**Conclusion:**

Patients below the recommended age for screening colonoscopy might be at increased risk for advanced CRC. There is need to decrease the recommended age for screening colonoscopy to prevent CRC or enable an early diagnosis in patients below 55 years.

## Background

Colorectal cancer (CRC) is one of the most common solid malignancies in western nations [1, 2]. Generally, CRC arises from a sequence of events that have been described as adenoma-carcinoma sequence [3]. This sequence can be effectively disrupted via screening. Preventing the transformation from an adenoma to a carcinoma, therefore, must be considered a priority for screening. The currently recommended CRC screening tests can be divided into two groups [2, 4, 5]. The first group of tests depends primarily on the detection of cancer in stool including the guaiac-based fecal occult blood test (gFOBT), the fecal immunochemical tests (FIT) based on fecal occult blood tests (FOBTs), and test for exfoliated DNA in stool (sDNA) [6, 7]. The second group enables the visualization of cancer and precancerous lesions via endoscopic (colonoscopy, sigmoidoscopy, capsule endoscopy) or radiologic (computed tomography, colonography, and barium enema) examination [8–10].

In Germany, screening colonoscopy was introduced in October 2002 as an alternative to FOBT for the screening of individual aged 55 years and above. The cost of screening colonoscopy for this population is fully covered by the statutory health insurance (SHI) which is responsible for insurance coverage of about 90% of the German population [11]. Screening colonoscopy for asymptomatic patients < 55 years, however, is not submitted to SHI reimbursement.

The available literature suggests an increase in the rate of CRC in young individuals without predisposition for CRC [12–15]. Our clinical experience suggests that a large portion of young individuals presents with large tumors and are diagnosed at an advanced stage. A tempting hypothesis is that this trend could be altered if the cost of screening colonoscopy in young individuals without predisposition to CRC were covered by the SHI. This study aimed at examining the clinicopathological characteristics and outcomes of CRC in patients below the recommended colonoscopy age.

## Methods

An analysis of prospectively collected data from our institutional CRC database was performed. Following the diagnosis of CRC, a written consent was received from each patient or their legal representative for the use of their data and specimens for research purposes. Ethics approval was received from the Ethics Committee at the Witten-Herdecke University. Data of all consecutive patients diagnosed with CRC was prospectively put into this database by specially trained individuals and study nurses. The database is continuously updated with information on the current status of registered patients. The fullness and accuracy of data is periodically controlled by an external audit. The database contains documentation of all cases of CRC irrespective of treatment option (endoscopic, palliation, or curative).

In our department, radical oncologic resection is performed in all patients undergoing curative surgery for CRC. Radical resection is reflected by the extent of surgical dissection of mesenteric lympho-vascular pathways including total mesocolic excision (CME) for colon cancer and total mesorectal excision (TME) for rectal cancer as described elsewhere [16, 17]. All procedures were performed either by an experienced senior surgeon or by a fellow/junior surgeon under direct supervision by a senior surgeon. The final tumor stage was reported using both the AJCC TNM and the Union Internationale Contre le Cancer (UICC) staging systems following histopathology. Patient characteristics including sex, age, and diagnosis were registered. Tumor variables included postoperative AJCC TNM tumor stage (pT), nodal stage (pN), and tumor location [18, 19].

Only patients undergoing elective curative surgery following colonoscopy and histopathological confirmation of CRC were included for analysis. Patients with multiple cancers were excluded from analysis. Patients with palliation procedures and cases with emergency procedures were excluded from the study.

To achieve our goal, the study population was divided into two groups using the age for recommended screening colonoscopy as cutoff. Thus, the study group included all patients < 55 years (screening colonoscopy generally not recommended) while the control group was made up of patients ≥ 55 years.

### Statistical analysis

The data generated was analyzed using the Statistical Package for Social Sciences (SPSS) version 23 (IBM Corp., Armonk, NY, USA). Continuous variables were described using medians and interquartile ranges (±) where necessary. The chi-square test was used to study the differences amongst both groups. Binary logistic regression analysis was used to calculate odds ratios (OR). The two-sided *p* values were reported where necessary with the level of significance set at *p* < 0.05. A 95% confidence interval (CI) was employed for all analyses. Survival diagrams were generated using the Kaplan-Meier Curve. The primary outcome was overall survival.

## Results

**Fig. 1:**
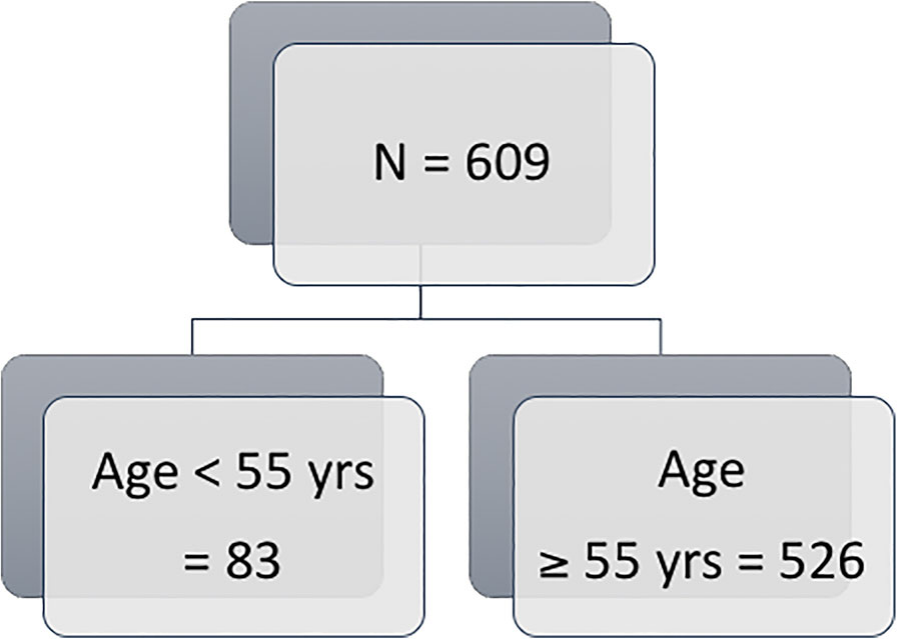
Distribution of study population

The study population consisted of 609 patients with CRC managed with radical surgery within a 5-year period from 2009 to 2013. Using the cutoff age for full insurance reimbursement for screening colonoscopy in Germany (55 years), the study population was divided into two age-dependent groups: < 55 years (study group) and ≥ 55 years (control group), Fig. 1. The median age of the study group was 48.0 ± 10 years (range 36–54 years) and 75.5 ± 8.3 years (range 55–87 years) for the control group. The baseline and clinicopathological features of the study population are presented in Table 1. Both groups were comparable with regard to gender distribution.

**Table 1:** Baseline and clinical characteristics of the study population

Characteristics	Age < 55	Age ≥ 55	*P* value
N	83	526	/
Sex			
Female	42 (50.6%)	230 (43.7%)	0.24
Male	41 (49.4%)	296 (56.3%)	
Mean age	47.6 ± 5.1 years	72.2 ± 8.5 years	/
Location			
Right colon	19 (22.9%)	164 (31.2%)	/
Transverse	5 (5.0%)	31 (5.9%)	
Left Colon	23 (27.7%)	165 (31.4%)	
Upper rectum	7 (8.4%)	40 (7.6%)	
Mid rectum	12 (14.5%)	60 (11.4%)	
Lower rectum	17 (20.5%)	66 (12.5%)	
AJCC tumor stage (pT)			
1	5 (6.0%)	83 (15.8%)	0.023
2	17 (20.5%)	96 (18.3%)	
3	43 (51.8%)	266 (50.5%)	
4	18 (21.7%)	81 (15.4%)	
AJCC nodal stage (pN)			
0	38 (45.8%)	319 (60.7%)	0.03
1	30 (36.1%)	120 (22.8%)	
2	15 (18.1%)	87 (16.5%)	
UICC			
I	11 (13.3%)	147 (28.0%)	0.03
II	25 (30.1%)	148 (28.1%)	
III	23 (27.7%)	129 (24.5%)	
IV	24 (28.9%)	102 (19.4%)	

The location of CRC was similar in both groups, Table 1. Advanced CRC (T3 and T4) were found significantly more often in the group < 55 years compared to the control group, 73.4 vs. 65.9%, *p* = 0.023. Nodal involvement was recorded in 54.2% of cases in the group < 55 years compared to 39.3% of cases in the control group. This difference was statistically significant, *p* = 0.03. Similarly, advanced CRC defined as stage III (27.7 vs. 24.5%) and IV (28.9 vs. 19.4%) per UICC staging criteria was seen significantly more often in the younger group compared to the control group, *p* = 0.03.

**Fig. 2:**
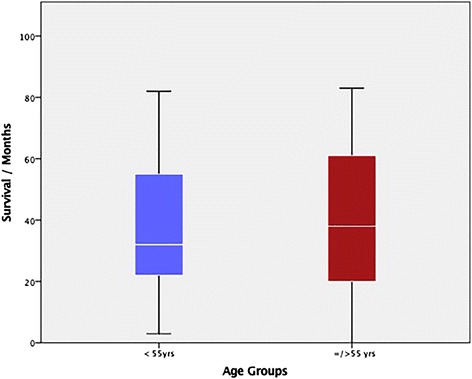
Median survival in both groups

**Fig. 3:**
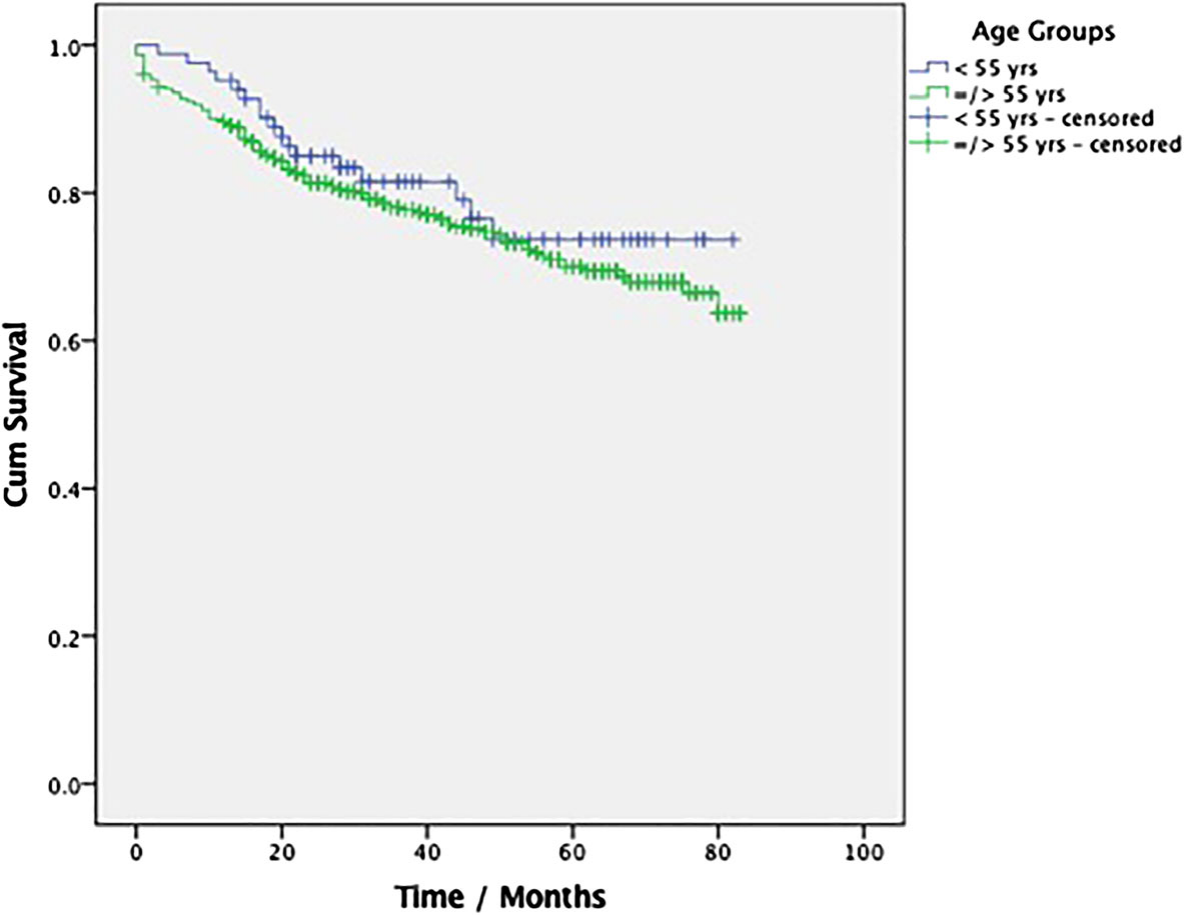
Kaplan Meier Curve showing the cumulative survival

The median overall survival was 46.0 ± 33.0 months (range 19–73 months) in the group < 55 years and 31.0 ± 27.0 years (range 10–82 months) in the control group, Fig. 2. This difference was not statistically significant, *p* = 0.61. Patients in the group < 55 years had a higher odd for advanced CRC (OR 1.67 (1.045–2.66), CI: 95%, *p* = 0.034). Nine cases (10.8%) of cancer-related death were recorded in the group < 55 years while 66 cancer-related (12.5%) deaths were recorded in the control group within the time examined. The cumulative survival in this study is presented in Fig. 3. There was no statistically significant difference in the rate of cancer-related death amongst both groups, *p* = 0.61.

## Discussion

Colorectal cancer is the second most common cause of cancer-related death in developed nations. The incidence of CRC has been shown to increase with age [20, 21]. Screening colonoscopy might detect colorectal polyps in asymptomatic individuals which are usually removed thereby disrupting the adenoma–carcinoma–sequences [5, 22]. Besides, screening colonoscopy in asymptomatic individuals also enables the diagnosis of CRC in an early stage for which curative resection is possible. Although the current German guidelines recommend screening for CRC in asymptomatic individuals beginning at the age of 50 years, full insurance reimbursement for screening colonoscopy is only offered to average-risk individuals aged ≥ 55 years [11]. Our clinical experience suggests that a sizeable portion of young patients (< 55 years) presenting with CRC is diagnosed with advanced tumors. We hypothesized that this trend could be changed if full reimbursement for screening colonoscopy were accessible for individuals < 55 years. This study examined the clinicopathological findings and outcomes of CRC in young patients < 55 years.

Prospectively collected data for a continuously updated colorectal database at the Helios University Hospital in Wuppertal, Germany, was retrospectively analyzed. Six hundred and nine surgically managed cases of CRC including 83 patients aged < 55 years were analyzed. Advanced local tumors pT3/T4 and nodal involvement (pN+) were seen significantly more often in patients < 55 years compared to the control group. The median overall survival was longer in the group with young patients in comparison to the control group. Equally, the rate of cancer-related mortality was lower in the group with young patients compared to the control group. However, there was no statistically significant difference amongst both groups with regard to cumulative survival.

Although screening for CRC is generally recommended in asymptomatic average-risk individuals starting at the age of 50 years, the screening options vary widely [22]. Amongst all screening options, colonoscopy has been proven to be most effective. Besides being a screening tool, colonoscopy is useful for the diagnosis of CRC by providing biopsies for histopathology. Furthermore, colonoscopy plays a key role in the prevention of CRC by disrupting the adenoma-carcinoma-sequence via removal of colorectal polyps. However, the risk of CRC has been thought to be low in asymptomatic individual < 55 years without hereditary predisposition. More so, the risk of colonoscopy-associated complications in this subpopulation is thought to overweigh its benefit [14]. Thus, colonoscopy is not generally recommended for screening young asymptomatic individuals at average-risk for CRC.

Current literature suggests a 3.4% decrease in CRC incidence and a 3.0% reduction in the rate of CRC-related mortality from 2003 to 2007 in patients > 50 years in the USA [23]. This trend has been attributed to increased screening, improvement of risk factors, and improved treatment of CRC [24]. In contrast to older individuals, the incidence of CRC in young adults with an average risk for CRC has been rising [12–15]. This trend must be blamed on the failure to detect and remove precancerous lesions in young adults due to lack of screening.

In Germany, colonoscopy has been established as the standard method of screening for CRC [25]. However, full insurance reimbursement for screening colonoscopy for individuals with an average risk for CRC is only possible at the age of 55 years and above. The costs for screening colonoscopy for average-risk individuals below this age limit are not reimbursed. Since there is a clear sequence of transformation from polyp (adenoma) to CRC, it appears logical that CRC in young individuals could be preventable or early detected, if the costs for screening colonoscopy in this subgroup were to be reimbursed by the SHI.

In the present study, advanced CRC was found significantly more often in young adults below the recommended colonoscopy age compared to controls. This is not surprising since these patients underwent colonoscopy because of abdominal symptoms secondary to advanced CRC. Similar results have been published previously [26–29].

CRC at a young age (< 50 years) should prompt the search for inherited high-risk CRC syndromes such as familial adenomatous polyposis (FAP), Lynch syndrome (LS), MUTYH-associated polyposis (MAP), etc. However, inherited syndromes have been seen in a minority of cases, and merely about 10% of CRC in young individuals has been attributed to hereditary conditions [14, 30–33]. The significance of identifying a hereditary syndrome as the cause of CRC must be the subsequent screening and genetic counseling of the patient’s family with the aim of identifying carriers of the defective gene in order to ensure a strict screening and eventually enable an individualized syndrome-dependent management concept. An interesting finding in our study was the age of presentation. The median age of presentation was 48 years. Unfortunately, screening for hereditary syndromes was not systematically performed in these patients at that time. This practice has changed, and all patients with CRC now undergo genetic screening for hereditary CRC-associated syndromes.

Despite the presence of advanced CRC at the time of diagnosis, the median survival was better in the young group compared to the control. This finding might be secondary to the absence of concomitant medical conditions in the young group. Besides, this survival trend might be a result of improved surgical and oncologic management. More so, the better survival advantage seen in younger patients has been attributed to a more aggressive adjuvant chemotherapy in young patients due to the assumption that early onset of CRC is a poor prognostic factor [34].

Some limitations of this study need to be addressed. First, the retrospective design must be seen as a limitation to this study. Genetic assessment to investigate a possible association with hereditary syndromes was not systematically performed and therefore could not be analyzed. This is a major limitation in light of the median age (48 years) of the study population. Second, data on the use of other screening options especially FOBT was not available. Although this screening option is not very reliable, it still plays an important role as an initial screening tool for CRC. Third, while the patients in the young group underwent colonoscopy due to symptoms, data on the portion of patients who underwent colonoscopy in the control group due to symptoms was not available and therefore could not be analyzed. Fourth, the effect of adjuvant chemotherapy was not analyzed. Finally, both groups were not matched with regard to BMI, smoking habits, and other sedentary lifestyles that might influence the development of CRC. Thus, there is a need for more investigation with better design and protocol.

Despite the above limitations, the results of this study confirm that young patients below the recommended colonoscopy screening age of 55 years in Germany are at increased risk for advanced CRC. This observation is in accordance with the result of a recently published population-based study from the Surveillance Epidemiology and End Results (SEER) database in the USA by Abdelsattar et al. [35].

Taken together, the results of this study are in line with available literature with regard to an increasing incidence of CRC in patients below the recommended screening age. Due to lack of screening, CRC is usually diagnosed in such cases at an advanced stage. This trend must be interpreted as an argument to re-think the starting age for CRC screening. Lowering the age limit for SHI reimbursement for screening colonoscopy in Germany might enable the prevention or early detection of CRC in young patients.

## Conclusion

The incidence of CRC in young patients at average-risk for CRC is increasing. In Germany, screening colonoscopy is not reimbursed for asymptomatic individuals at average-risk for CRC below the age of 55 years. For such individuals, colonoscopy is usually performed due to symptoms and CRC is frequently diagnosed in an advanced stage. Lowering the age limit for insurance reimbursement for screening colonoscopy in Germany might enable the prevention or an early detection of CRC in young patients.

## Abbreviation

AJCC: American Joint Committee of Cancer
CRC: Colorectal cancer
FAP: Familial adenomatous polyposis
FIT: Fecal immunochemical tests
gFOBT: Guaiac-based fecal occult blood test
LS: Lynch syndrome
MAP: MUTYH-associated polyposis
SEER: Surveillance Epidemiology and End Results
UICC: Union Internationale Contre le Cancer
